# Beat-to-Beat Blood Pressure Estimation by Photoplethysmography and Its Interpretation

**DOI:** 10.3390/s22187037

**Published:** 2022-09-17

**Authors:** Vincent Fleischhauer, Aarne Feldheiser, Sebastian Zaunseder

**Affiliations:** 1 Faculty of Information Technology, University of Applied Sciences and Arts Dortmund, 44139 Dortmund, Germany; 2 TU Dresden, Institute for Biomedical Engineering, 01069 Dresden, Germany; 3 Department of Anaesthesiology, Intensive Care Medicine and Pain Therapy, Evang. Kliniken Essen-Mitte, Huyssens-Stiftung/Knappschaft, 45136 Essen, Germany; 4 Department of Anaesthesiology and Operative Intensive Care Medicine (CCM, CVK), Charité—Universitätsmedizin Berlin, Corporate Member of Freie Universität Berlin, Humboldt-Universität zu Berlin, and Berlin Institute of Health, 13353 Berlin, Germany

**Keywords:** blood pressure, pulse wave decomposition, pulse decomposition analysis, photoplethysmography, Shapley values, estimation, interpretation, explainability

## Abstract

Blood pressure (BP) is among the most important vital signals. Estimation of absolute BP solely using photoplethysmography (PPG) has gained immense attention over the last years. Available works differ in terms of used features as well as classifiers and bear large differences in their results. This work aims to provide a machine learning method for absolute BP estimation, its interpretation using computational methods and its critical appraisal in face of the current literature. We used data from three different sources including 273 subjects and 259,986 single beats. We extracted multiple features from PPG signals and its derivatives. BP was estimated by xgboost regression. For interpretation we used Shapley additive values (SHAP). Absolute systolic BP estimation using a strict separation of subjects yielded a mean absolute error of 9.456mmHg and correlation of 0.730. The results markedly improve if data separation is changed (MAE: 6.366mmHg, *r*: 0.874). Interpretation by means of SHAP revealed four features from PPG, its derivation and its decomposition to be most relevant. The presented approach depicts a general way to interpret multivariate prediction algorithms and reveals certain features to be valuable for absolute BP estimation. Our work underlines the considerable impact of data selection and of training/testing separation, which must be considered in detail when algorithms are to be compared. In order to make our work traceable, we have made all methods available to the public.

## 1. Introduction

Blood pressure (BP) is one of the most important vital signs. It has high relevance in a variety of clinical and out-of-hospital applications. Invasive methods are the gold standard for BP measurement. Such methods are restricted to clinical environments and include discomfort as well as a risk for infections and, in case of disconnection, serious patient harm. Sphygmomanometry is the most common type of BP measurement [1]. While being non-invasive, sphygmomanometry is an intermittent measurement technique. It thus prevents beat-to-beat analyses and cannot capture the dynamic characteristics of BP [2]. Non-invasive methods such as the volume clamp method or applanation tonometry provide continuous measurements but have known limitations, e.g., regarding their robustness, carry risk for venous congestion or are sensitive to imprecise placements. Further, (commercial) measurement equipment is bulky and expensive [3,4,5,6].

Surrogate approaches provide a user-friendly alternative to assess BP, typically on a beat-to-beat basis [7]. Surrogate approaches do not measure BP but estimate BP from a single or a set of variables. The most widespread surrogate approach exploits the relationship of pulse wave velocity (PWV) (or pulse transit time (PTT) or pulse arrival time (PAT), respectively, [7,8,9,10]) and BP. PWV is estimated from time differences of proximally and distally recorded cardiovascular effects, most often from electrocardiogram (ECG) and distal photoplethysmography (PPG) [7,11]. PWV methods typically invoke initialization, i.e., a single or repeated cuff measurement to yield absolute BP. Afterwards, beat-to-beat PWV is converted into a beat-to-beat BP estimate. Most approaches make some physical assumption that can formulate the relation between PWV and BP mathematically, e.g., a linear or logarithmic dependency.

In recent years, the number of surrogate approaches that use machine learning (ML) for BP estimation increased drastically [12]. Owing to its good availability and high user comfort, the vast majority of such works use PPG. These works employ variable input information from PPG and a variety of ML techniques. Input information is represented by discrete features or complete signal excerpts (end-to-end learning), which can originate from the PPG, from its derivative(s) or from pulse wave decomposition (PWD). ML techniques range from trees and forests [13,14] over support vector machines [15,16] to (deep) neural networks [17,18,19,20,21]. Remarkably, while early surrogate approaches most often invoke initialisation and track changes in BP, an increasing number of works aim at absolute BP estimation from PPG. In fact, a reliable method for absolute BP estimation solely using PPG would have huge impact to various medical fields and is thus of immense interest.

However, the published results on absolute BP estimation vary considerably. Moreover, the function of the proposed models can hardly be explained because the input dimension typically is high and most employed models are black boxes such as CNN and LSTM [22]. In terms of medical usage and further development, explainability is considered highly important [23,24]. In different fields it has gained attention [25,26,27]; however, to the best of our knowledge, no works have investigated the explainability of ML models in the field of BP estimation.

Our work therefore aims at a PPG-based ML method for absolute BP estimation and its explanation using computational methods. Such content is accompanied by a critical appraisal of recent approaches for absolute BP estimation in face of the employed training/testing strategy. In order to be comparable to the literature, we do not propose a novel algorithm for BP estimation but adopt a feature-based method for BP estimation recently proposed by Hu et al. [13]. Hu et al.’s work uses multiple common features including PWD together with an ensemble regressor. It yields highly accurate results and it is representative of many current works in the field. We complement the proposed approach with features from second derivative and carry out an in-depth analysis of feature contribution by means of Shapley values in order to explain the model’s function. Note that the following consideration primarily focus on systolic BP as it is the most commonly estimated type of BP. However, our discussion also contains some remarks on diastolic BP.

The remainder of this work is structured as follows. Section 2 provides an overview of the feature-based methods for absolute BP estimation using PPG. Section 3 describes the used data, feature extraction and estimation models. The results from Section 4 are then discussed in Section 5. Finally, in Section 6, we provide an outlook for future work.

## 2. State of the Art

There are numerous works that focus on the estimation of BP with PPG [11,28,29,30,31]. In this work we concentrate on feature-based methods for absolute BP estimation using PPG. Table 1 provides on overview of such works. We included publications that do not use modalities other than a single PPG and do not use initialization (i.e., focus on absolute BP estimation). The overview only contains works that report the mean absolute error.

First, it should be noted that the comparability of the works is limited as they do not only use different approaches to BP estimation but also different datasets.

Notably, there are some works with exceptionally good prediction results (MAE <7mmHg). In most cases, these results are not comparable to the rest as there is a difference in the separation of data into test and training sets. In Table 1, we highlighted such differences in the ‘Clear Separation’ column, which indicates whether subjects were exclusively assigned to test and training data (‘yes’) or not (‘no’). As ML algorithms are thus able to learn on subjects of the test set, and predictions can improve considerably. Works with an MAE of below 7mmHg do not use clear separation with one exception being Jain et al. [41]. Jain et al. used their own data, which consist of photoplethysmography imaging (PPGI) measurements and a single cuff measurement of BP. The dataset contains 45 normotensive subjects with ages of between 20 and 40 years. In contrast, commonly used publicly available datasets such as the MIMIC database consist of continuous BP measurements of subjects of a more diverse age and BP spectrum, thus being far more difficult to predict. Though the results of Jain et al. are remarkable (keeping in mind the non-contact approach), the data characteristic are likely to contribute to the comparatively low prediction error despite their data being clearly separated.

Otherwise, errors close to 10mmHg are common (which still does not fulfil the normative requirements on BP estimation). A commonality of these works is the absence of reasoning for the used features. Besides the work of Gaurav et al. [42] and Hasanzadeh et al. [34], all works either only state that they used features that are used in literature to estimate BP or do not state any reason for their feature selection. Gaurav et al. explain the physiological information contained in used feature classes. Hasanzadeh et al. state the physiological correlates for some of their features.

## 3. Methods And Materials

### 3.1. Data

For our analysis we used three datasets that contain PPG signals and blood pressure measurements. The following paragraphs provide a brief overview of the data.

#### 3.1.1. CPT

The first dataset contains data from 22 healthy subjects (age 25.5±3.73 years, 10 female) during a cold pressure test (CPT). The study was approved by the Institutional Review Board of the TU Dresden (EK119032016). All subjects provided their written informed consent. The subjects were included twice, once in a sitting position and once in a supine position. We discarded one recording due to technical problems. Thus, the dataset contains 43 usable records. After an initial resting phase of 8 min, the subjects immersed their hand into cold water ( 3 ${}^{\circ }C$). The immersion lasted for 3 min, but subjects were allowed to quit earlier. CPT, in general and within the experiment, leads to an instantaneous increase in blood pressure [46]. After the immersion, the subjects remained rested for another 21 min. The data consists of non-invasive continuous BP measurements (Finometer Midi, Finapres Medical Systems) and finger PPG signals recorded from the non-immersed hand with a sampling frequency of 1000 Hz [46].

#### 3.1.2. PPG-BP

The publicly available PPG-BP database [47] contains records from 219 healthy and non-healthy subjects (age 57.17±15.87 years, 115 female). The experiment comprised an initial resting phase of 10 min and a 3 min measurement phase without any applied stimuli. Each record contains one systolic and diastolic blood pressure measurement that represents the BP for the whole measurement phase as well as three PPG segments, each with a duration of 2.1 s. The PPG signal was measured at a sample rate of 1000 Hz (SEP9AF-2, SMPLUS Company, Seoul, South Korea) at the fingertip of the left index finger. The BP sensor (Omron HEM-7201, Omron Company, Kyoto, Japan) was attached to the right forearm [47].

#### 3.1.3. Queensland

As the third dataset, we included the University of Queensland Vital Signs Dataset, which consists of 32 records (gender of subjects not stated) with a duration ranging from 13 min to 5 hours (median 105 min). The data were collected from subjects under anesthesia. The data were recorded using multiple devices (Philips IntelliVue MP70 & Philips IntelliVue MP30, Philips Healthcare, Amsterdam, Netherlands; Datex- Ohmeda Aestiva/5, GE Healthcare, Chicago, IL, USA) and contain PPG waveforms at a sample rate of 100 Hz and non-invasive BP measurements [48].

### 3.2. Preprocessing

We filtered the PPG signals with a bandpass filter (5th-order Butterworth filter with cut-off frequencies of 0.4 Hz and 12 Hz). Single beats from the PPG signals were detected with the method of Lazaro et al. [49], which considers the steepest ascent as the detection point. We then segmented each beat by detecting the minima in the segments before and after the detection point. The segmentation yielded 72,106 beats for the CPT dataset, 254,609 beats for the Queensland dataset and 1125 beats for the PPG-BP dataset.

**Figure 1:**
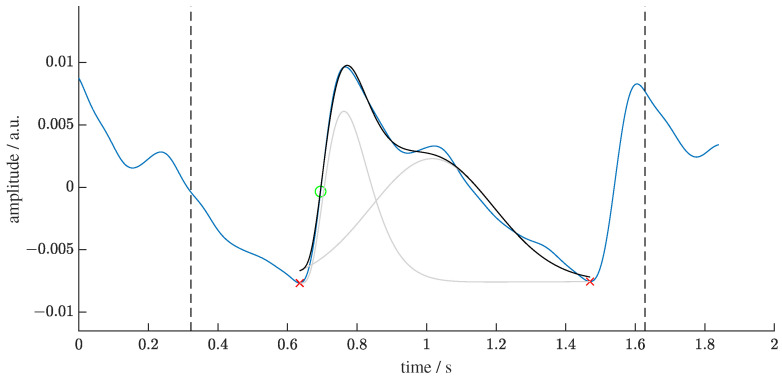
Visualization of the segmentation and decomposition of a PPG beat. The green circle marks the detection point from the algorithm of Lazaro et al. [49]. The black dashed lines display the interval around the detection point in which the minima (red crosses) are searched. The beat between these minima is then decomposed into two kernels (light grey). The sum of the kernels is the recomposed beat (black line).

We removed linear trends and normalized each beat to within the range of zero to one. We then applied PWD to each single beat. The aim of PWD is twofold: to yield decomposition parameters and to denoise by performing recomposition. We used the GammaGaussian2 decomposition algorithm (i.e., decomposition by a Gamma Kernel and a Gaussian Kernel, see Figure 1) that was described previously [50]. A reconstructed beat *y* for the Gamma–Gaussian algorithm with 2 kernels can be described as:

(1) $$y_{\mathrm{GammaGaussian}2}\left(t,\theta \right)=\frac{\beta_{1}^{\alpha_{1}}}{s_{1}\;\cdot \;\Gamma \left(\alpha_{1}\right)}t^{\alpha_{1}-1}e^{-\beta_{1}t}+a_{2}\;\cdot \;e^{\left(\frac{-{\left(t-\mu_{2}\right)}^{2}}{2\sigma_{2}^{2}}\right)}\;.$$

Each reconstructed beat is a function of time *t* and an optimization vector θ=[a,μ,σ]. The interior point optimization algorithm fits the kernels to the PPG beats using the following constraints:

(2) $$\begin{array}{cc} a_{1} & >a_{2} \end{array}$$

(3) $$\begin{array}{cc} \mu_{1} & <\mu_{2} \end{array}$$

The initial values for the algorithm are explained in detail in [50]. Figure 1 displays the processing of the PPG signals.

### 3.3. Feature Extraction

As stated before, we adopted features used by Hu et al. [13]. Additionally, we included the feature b/a, i.e., the relation of the *b* peak to the *a* peak of the second derivative, to assess the benefit of a second derivative analysis. Accordingly, we extracted the following four types of features from the PPG beats: PWD, second derivative, statistical, and frequency features. The used features are listed in Table 2.

PWD yields the parameters of the kernels it decomposes the beat into. Some works assess the relationships between the kernels [51,52]. We used these parameters as features without prior combination in accordance to Hu et al. [13].

The analysis of the second derivative of a PPG beat evaluates the ratios of the characteristic peaks. We included b/a as this is the most commonly used feature of the second derivative in BP estimation [42,53,54]. The second derivative is calculated from the reconstructed beat to reduce the impact of noise.

Statistical features assess the general shape of the PPG beat. As in previous works, we included standard deviation (SD), kurtosis (kurt) and skewness (skew). All statistical features are calculated from reconstructed beats [13,42,55].

We calculated the statistical features for a PPG beat *p* with *N* samples and its mean $\bar{p}$ as follows:

(4) $$\mathrm{SD}=\sqrt{\frac{1}{N}\sum_{n=1}^{N}{\left(p_{n}-\bar{p}\right)}^{2}}$$

(5) $$\mathrm{kurt}=\frac{\frac{1}{N}\sum_{n=1}^{N}{\left(p_{n}-\bar{p}\right)}^{4}}{{\left(\frac{1}{N}\sum_{n=1}^{N}{\left(p_{n}-\bar{p}\right)}^{2}\right)}^{2}}$$

(6) $$\mathrm{skew}=\frac{\frac{1}{N}\sum_{n=1}^{N}{\left(p_{n}-\bar{p}\right)}^{3}}{{\left(\sqrt{\frac{1}{N}\sum_{n=1}^{N}{\left(p_{n}-\bar{p}\right)}^{2}}\right)}^{3}}\;.$$

Like Hu et al. [13], we also included the fundamental frequency and the first to third harmonic for the BP estimation. Thereto, we extended each original beat to ten copies of itself and Fourier transformed that signal.

### 3.4. Estimation Models

We used the python library xgboost [56] to estimate the blood pressure as suggested in the work of Hu et al. [13]. We randomly selected 80% of the subjects of each dataset for training (231,997 samples) and used the remaining 20% for testing (27,989 samples). Both measurements of the CPT subjects were assigned to the same set (test or training). We thus implemented a strict separation of test and training data on a subject level. For the training of the model, we used all 15 features of Table 2 as inputs and SBP as the response variable. To account for the imbalanced distribution of training and test data, we applied sample weights (all samples with SBP above half the maximum BP value in the dataset were weighted with 0.375). We used a median filter with a kernel size of 11 for the CPT and Queensland dataset on the prediction and ground truth. We did not filter the PPG-BP dataset as there were too few samples per subject. We did not attempt hyperparameter optimization as the focus of this work is the explanation of the model rather than the accuracy of the prediction.

### 3.5. Evaluation

The evaluation has two parts. First, we evaluate the model quality concerning absolute blood pressure estimation to show that our model performs comparably to similar ones in the literature. Secondly, we evaluate the impact of features on the prediction to interpret the model’s function.

#### 3.5.1. BP Estimation

In order to assess our model’s quality, we used the mean absolute error (MAE), mean error (ME), standard deviation of the error (SDE) and Pearson correlation coefficient (*r*) from the *N* true BP values *x* and the estimated BP $\hat{x}$ from the test data according to:

(7) $$\mathrm{MAE}=\frac{1}{N}\sum_{n=1}^{N}\left|x_{n}-{\hat{x}}_{n}\right|$$

(8) $$\mathrm{ME}=\frac{1}{N}\sum_{n=1}^{N}\left(x_{n}-{\hat{x}}_{n}\right)$$

(9) $$\mathrm{SDE}=\sqrt{\frac{1}{N}\sum_{n=1}^{N}{\left(\left(x_{n}-{\hat{x}}_{n}\right)-\bar{x-\hat{x}}\right)}^{2}}$$

(10) $$r=\frac{\sum_{n=1}^{N}\left(x_{n}-\bar{x}\right)\left({\hat{x}}_{n}-\bar{\hat{x}}\right)}{\sqrt{\sum_{n=1}^{N}{\left(x_{n}-\bar{x}\right)}^{2}{\left({\hat{x}}_{n}-\bar{\hat{x}}\right)}^{2}}}\;.$$

#### 3.5.2. Shapley Values

The basic idea of explainable machine learning based on Shapley values is to compute the average marginal contribution of a feature value across all possible coalitions, i.e., sets of features [57]. Shapley values represent the impact of a feature on the prediction of a model for a given input. They can be computed using a weighted sum that represents the impact of each feature being added to the model averaged over all possible orders of features being introduced [58]:

(11) $$\phi_{j}\left(f,x\right)=\sum_{S\subseteq S_{\mathrm{all}/j}}\frac{|S|!(M-|S|-1)!}{M!}\left[f_{x}\left(S\cup j\right)-f_{x}\left(S\right)\right]$$

In (11), $\phi_{j}\left(f,x\right)$ represents the Shapley value for feature *j* of the prediction f(x) for sample *x*, *M* is the number of features and *S* is the subset of input features that are present in the prediction.

We used the python library SHAP (SHapley Additive exPlanations) that efficiently implements this concept from game theory for certain machine learning models by computing SHAP values [59]. In SHAP, the explanation is represented as an additive feature attribution. This means that the prediction $g(x_{i})$ for a sample $x_{i}$ can be represented as a linear model:

(12) $$g\left(x_{i}\right)=\phi_{0}+\sum_{j=1}^{M}\phi_{j}^{\left(i\right)}$$

In (12), the prediction is represented as the sum of $\phi_{0}$, which is the average prediction, and the sum of the SHAP values ϕ of all *M* features for the *i*th sample.

To compute a global SHAP value over all *N* samples, we use the mean absolute of the SHAP values ϕ of each feature, which yields the importance $I_{j}$ for the *j*th feature:

(13) $$I^{\left(i\right)}=\frac{1}{N}\sum_{j=1}^{N}\left|\phi_{j}^{\left(i\right)}\right|$$

We also analyzed sample-wise SHAP values in relation to their feature values by creating a beeswarm plot to obtain an overview of the impact of the feature values on the prediction.

## 4. Results

**Figure 2:**
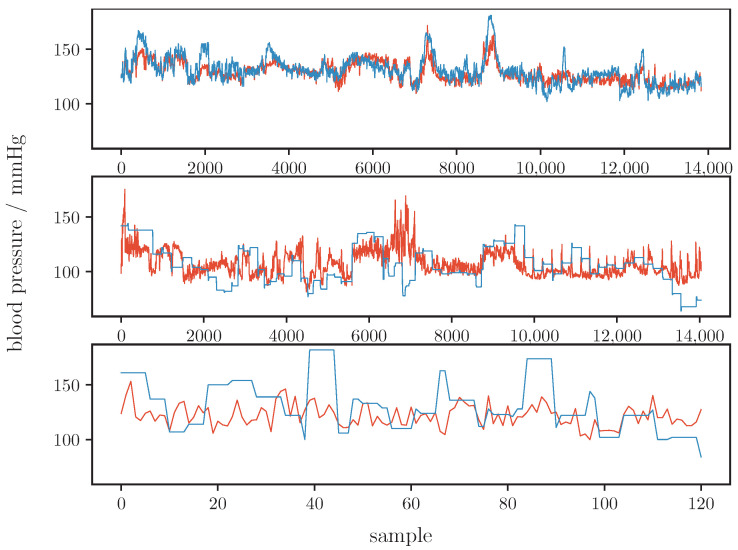
Comparison of estimated blood pressure (orange line) and ground truth (blue line) for all samples of the test set. The top plot shows the CPT data, the middle plot shows the Queensland data, the bottom plot shows the PPG-BP data.

Table 3 shows our models results for the whole dataset and for the three single datasets (CPT, Queensland and PPG-BP). Figure 2 illustrates the prediction of our model and the ground truth graphically. These results are comparable to that of other current works such as that of Zhang et al. [40] or Hasanzadeh et al. [34] (see Section 5 for a detailed analysis on the performance).

**Figure 3:**
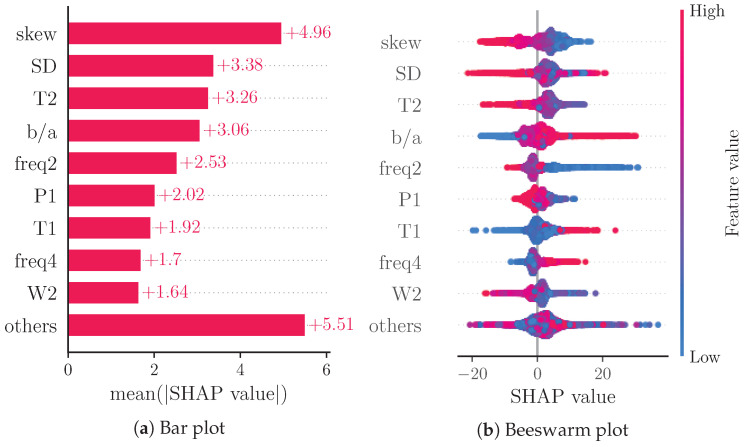
(**a**) shows a bar plot of mean absolute SHAP values that indicates global feature importance (mean absolute for each feature over all given samples). (**b**) depicts a beeswarm plot of SHAP values. For each sample, this plot shows a dot on each feature row. The features are ordered according to the mean absolute SHAP values for each feature. Depicted are the nine features with the highest mean absolute SHAP value and the sum of the remaining features.

Figure 3a reports a ranking of the mean absolute SHAP values. Notably, the four most important features are: skew (4.96), SD (3.38), T2 (3.26) and b/a (3.06). The beeswarm plot in Figure 3b shows the relationship of the feature values and the SHAP values for each feature with each dot representing the SHAP value of a feature for a prediction.

## 5. Discussion

### 5.1. Quality of Absolute BP Estimation

Compared to Hu et al., our results are markedly worse. At first glance, this is surprising as we reproduced Hu et al.’s work. Minor modifications relate to the integration of the second derivative and to the PWD (for PWD, we used two instead of three Kernels as we showed that algorithms with two kernels are more robust against noise but otherwise comparable [50]). Both modifications are not likely to degrade the results. However, as can be seen in Table 1, our results are comparable to those works that clearly separate between training and test subjects. Notably, if we apply the same training/testing strategy as Hu et al., i.e., if we do not strictly separate according to subjects, our results improve considerably and closely approach the results of Hu et al. (MAE: 6.366mmHg, ME: 2.886mmHg, SDE: 12.022mmHg, *r*: 0.874). Such an improvement is expected, but it underlines the importance of data separation towards an objective comparison of different works. Clearly, even data selection, i.e., the characteristics of data, heavily impacts the results. With respect to our data, there are large differences in the quality of BP estimation between data from different sources. Our model achieved the best results for the CPT data, while the predictions for the Queensland and PPG-BP data are markedly worse. The most likely reasons for the difference in quality are the subjects’ states and associated BP ranges. Table 4 shows the characteristics of BP for test and training subsets of all datasets used. From Table 4 and Figure 2 it is evident that the Queensland data contain comparatively low SBP samples (below 100mmHg), which is probably caused by anesthesia. Our model often overestimated BP (as indicated by a negative ME for the Queensland dataset). Further, the ground truth for the Queensland data does not seem to be recorded on a beat-to-beat basis. The authors of the study do not state a sampling rate for the non-invasive BP measurements but Figure 2 suggests intermittent BP readings. The reference BP thus neglects higher frequency variations and potentially introduces estimation inaccuracies. This problem is worse for the PPG-BP dataset. Here, BP is measured once per subject and for each subject three PPG excerpts of 2.1s duration, and each from a 10min time frame, are provide. Despite this obvious limitation, we included such data in our analysis as they are often used. However, as in the data separation process before, data selection has a critical impact on the results and must be carefully considered in comparisons of different works.

To allow meaningful comparisons and foster traceability, we included data from different origins, provided aggregated and separated results and make our sources freely available (source code available via https://github.com/vifle/ppgBP (accessed on 22 July 2022)). Overall, our results indicate the need for further improvements prior to potential clinical use. Such improvements do not only relate to data processing but should also invoke modifications to the frontend as suggested in the current work, e.g., measurement systems that are able to take the contact pressure between skin and sensor into account might add valuable information as the amplitude and morphology of the PPG vary with contact pressure [60]. Recently, Cao et al. presented a method to estimate the contact pressure by means of a single PPG sensor [61]. Another approach to enhance BP estimation in the future is to use multiple wavelengths and exploit the varying interactions of wavelengths with tissue [62]. However, notwithstanding such enhancements, taking into account the data selection and data separation, we can state that the proposed method yields state-of-the-art results on absolute BP estimation. This is a precondition for the meaningful interpretation and assessment of feature importance.

### 5.2. Model Interpretation

The considered features for absolute BP estimation in the literature typically originate from previous works that use these features for BP estimation. Most works included in Table 1, and on BP estimation in general, do not explain the relationship between the selected features and BP. A few of the works use large feature pools, some of which select a subset of them using varying criteria [35,39]. Hasanzadeh et al. explain some of their features’ physiological correlates [34]. Gaurav et al. state reasons for the types of features used [42].

Aside from Hu et al., other works do not use PWD features. Derivative features are used in only four of the works [35,37,39,42]. Our analysis of SHAP values shows that derivative and decomposition features are among the most important features of the model, thus indicating their importance in BP estimation. The feature importance of SD and skew is likely due to the fact that these features evaluate the shape of the PPG pulse as a whole.

**Figure 4:**
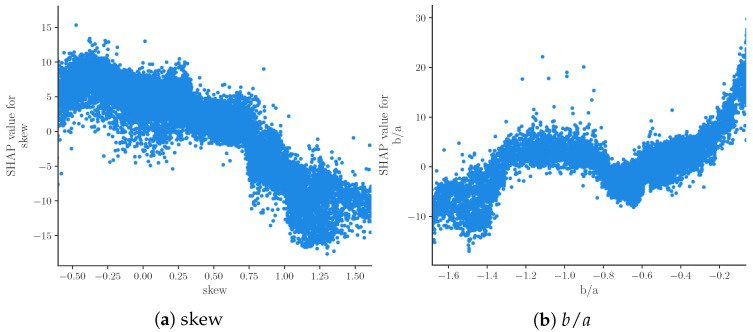
Dependence plot for features skew and b/a showing the relationship of the value of the feature and its SHAP value for all samples. All samples with feature values in the first and 99th percentile are removed.

Figure 3b shows the relation of feature values and SHAP values for the nine most important features. Due to the representation of predictions as an additive model of SHAP values, positive SHAP values can be associated with high BP predictions and negative SHAP values can be associated with low BP predictions. The beeswarm plot thus provides an impression of the relationship of feature values and BP prediction. Features b/a, T1 and freq4 seem to be positively correlated with SBP, while skew, T2 and W2 are clearly negatively correlated with SBP. The remaining features do not exhibit such clear relations. This could be caused by poor prediction or nonlinear relationships. The relationship of feature values and SHAP values can be analyzed in further depth by means of dependence plots. Figure 4 shows such a dependence plot for skew and b/a. When outliers (the first percentile and 99th) are removed, a clearly negative correlation between feature and SHAP values can be observed for skew (see Figure 4a), while b/a exhibits a clearly positive correlation (see Figure 4b). Nonlinear relationships could be caused by interaction effects between features. These effects are not analyzed in this work.

**Figure 5:**
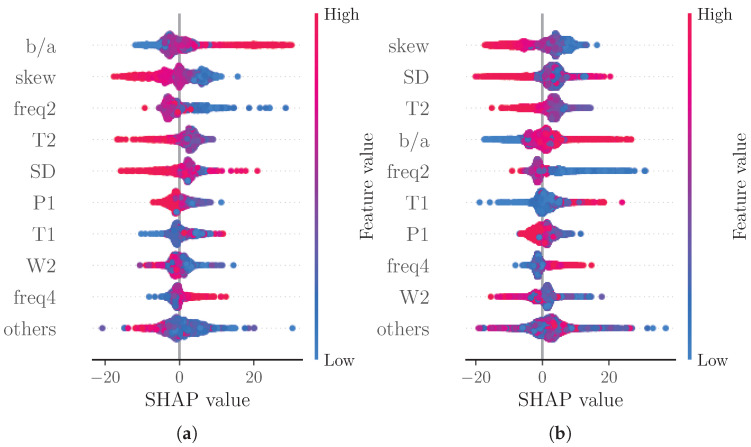
Beeswarm plot of SHAP values for subsets of samples of test set according to the MAE values of the prediction models. (**a**) error greater than two times the MAE, (**b**) error lower than half the MAE.

Note that native SHAP does not consider the quality of the prediction. We therefore also analyzed the SHAP values of subsets of the test data categorized into samples with an error greater than two times the MAE (see Figure 5a) and samples with an error lower than half the MAE (see Figure 5b). With this analysis, we assessed whether certain features significantly contribute to poor or good predictions, respectively. For the subset of good predictions, the four most important features remain unchanged. For the subset of bad predictions, however, the order of the most important features changes. The most important feature for this subset is b/a; additionally, freq2 supersedes T2 out of the four most important features. The increased importance of b/a could be explained by this feature’s susceptibility to errors. As this is a feature of the second derivative, even small changes to the shape of the beats’ rising slope due to decomposition and reconstruction can cause substantial errors in the feature value.

**Figure 6:**
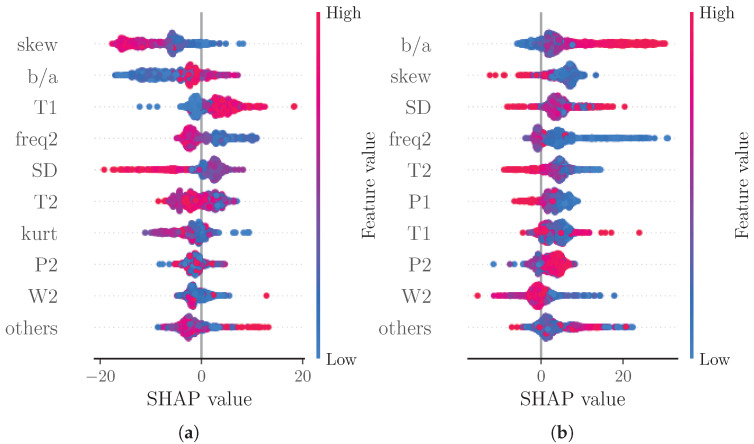
Beeswarm plot of SHAP values for subsets of samples of test set according to the predicted SBP. (**a**) prediction lower than 0.8 times the mean prediction, (**b**) prediction greater than 1.2 times the mean prediction.

Another analysis considered SHAP values separated into subsets of low BP predictions (0.8 times the mean prediction, see Figure 6a) and high BP predictions (1.2 times the mean prediction, see Figure 6b). As expected due to the additive nature of SHAP values, the majority of points in the beeswarm plots shifts towards negative SHAP values for low BP predictions and towards positive SHAP values for high BP predictions. For high BP predictions, freq2 becomes more important than T2. For low BP predictions though, T1 and freq2 become more important than T2 and SD.

**Figure 7:**
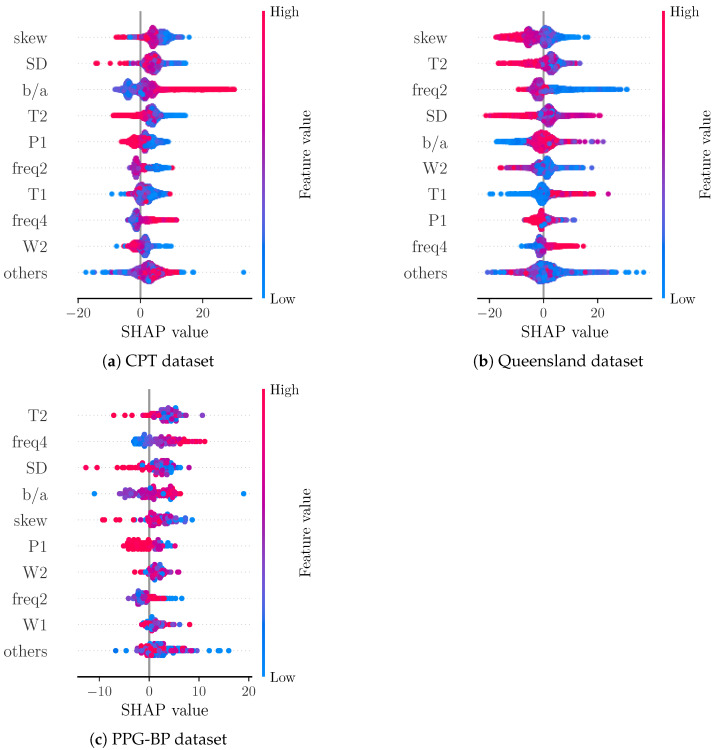
Beeswarm plot of SHAP values for subsets of samples of test set according to the database.

Figure 7 shows a comparison of SHAP values for the data from each included dataset separately. For CPT, the most important features remain unchanged. Interestingly, the apparently positive correlation of feature value and SHAP value for T1 from the overall analysis cannot be observed as clearly for the CPT subset. For the Queensland subset, T1 show a similar behaviour as in the overall analysis. Furthermore, the other subsets exhibit skew, T2, b/a and SD also as the most important features. Notably, b/a becomes the fifth most important feature for the Queensland subset, while freq2 is the third most important feature and freq4 is the second most important feature for PPG-BP with skew becoming the fifth most important feature. A difference between the datasets is the shape of the ground truth BP values (see Figure 2). The BP values of CPT fluctuate much more than those of Queensland. A possible explanation for the greater importance of b/a in CPT could be the ability of b/a to track small morphological changes that reflect small changes in BP. This is more important for CPT than for Queensland, as the BP for Queensland remains constant in larger segments compared to CPT. In PPG-BP, the number of samples is too low to reliably assess the relationship between feature and SHAP values.

### 5.3. Feature Importance in an Alternative Model

**Figure 8:**
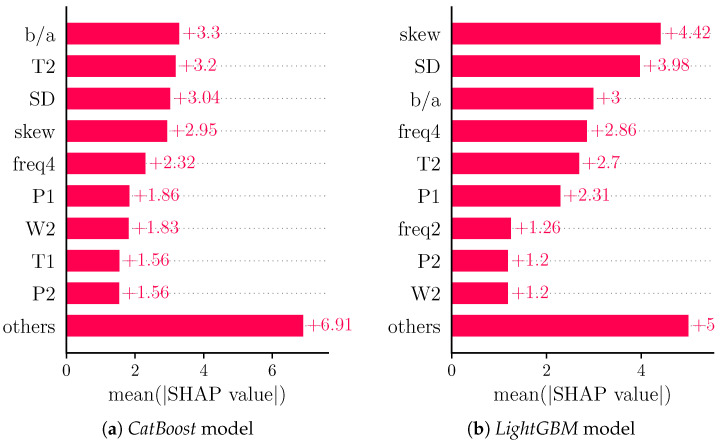
Bar plot of mean absolute SHAP values for comparison models. Shows global feature importance (mean absolute for each feature over all given samples).

In order to provide a more general view on feature importance than is achieved with a single model, we analyzed the feature importances for regression models generated by *CatBoost* [63] (Figure 8a) and *LightGBM* (Figure 8b) [64].

Both models exhibit different orders of features in terms of feature importance. Notably, the four most important features remain b/a, T2, SD and skew for the *CatBoost* model, whereas freq4 supersedes T2 in the *LightGBM* model, underlying the general relevance of such features.

### 5.4. Analysis with Respect to DBP

Our analysis primarily focused on SBP. It can, however, be readily applied to DBP. For illustration, we conducted some analyses on DBP. DBP yields an MAE of 7.1mmHg and ME of −0.214mmHg.

**Figure 9:**
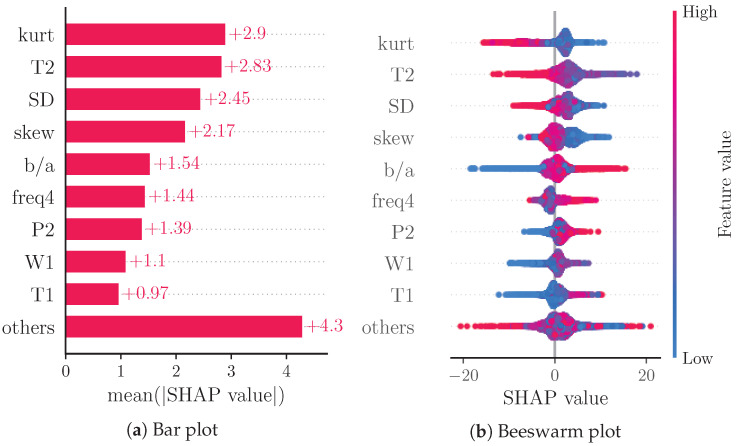
SHAP values for DBP prediction model on the whole dataset. (**a**) shows a bar plot of mean absolute SHAP values that indicates global feature importance (mean absolute for each feature over all given samples). (**b**) depicts a beeswarm plot of SHAP values. For each sample this plot shows a dot on each feature row. The features are ordered according to the mean absolute SHAP values for each feature. Depicted are the nine features with the highest mean absolute SHAP value and the sum of the remaining features.

Figure 9 shows the SHAP values for a DBP prediction model based on the same features as our SBP prediction model. The four most important features of the SBP model are ranked two to five for the DBP model. This shows the importance of general BP estimation of these four features. For this model, kurt becomes the most important feature. For the SBP overall model, this feature was not one of the nine most important features, but was relevant for SBP predictions of lower than 0.8 times the mean prediction (see Figure 6a). Notably, the SHAP values for the DBP model seem to be lower than those for the SBP model on average. This can be explained by the additive nature of SHAP values as DBP values are generally lower than SBP values.

## 6. Conclusions & Outlook

The presented work demonstrates one approach to interpreting the function of multivariate ML methods. Our results provide strong evidence of using features from PPG and its derivatives. Such finding should affect future methods on BP estimation using the PPG, which usually do not account for the selected features. Our considerations further highlight the immense impact of data selection and separation. In future works, further in-depth analyses should consider the interaction effects between features to develop a better understanding of the relationship between feature values and predictions.

## Figures and Tables

**Table 1:** Works regarding feature-based absolute BP estimation from PPG. Each entry reports the machine learning model, datasets, number of features and feature types used. The ‘Clear Separation’ column indicates whether the subjects are clearly separated into test and training data (‘yes’) or not (‘no’). Publications that do not declare their method of separation are labelled ‘unknown’. We show the best results achieved for each publication.

Author	Method	Datasets	Number of Features	Feature Types	Clear Separation	MAE_SBP_ ± SD_SBP_ in mmHg
[32]	Bi-GRU + GRU + attention	MIMIC II	22	original	unknown	2.58±3.35
[13]	XgBoost	MIMIC, Queensland, PPG BP	16	PWD, original, frequency	no	5.38±9.66
[33]	gradient boosting machine	HYPE, EVAL	21	original	yes	8.79±3.17
[34]	AdaBoost	UCI (MIMIC II)	19	original	yes	8.22±10.38
[35]	fully connected neural network	MIMIC II	32	original, first derivative, second derivative	no	3.21
[36]	LSTM	MIMIC II	7	original	unknown	3.25±4.76
[37]	random forest	MIMIC III	16	original, first derivative, frequency	yes	18.34
[16]	ANN, SVR	MIMIC II	21	original	unknown	1.21
[38]	multilayer perceptron (ANN)	MIMIC	22	original, frequency	no	4.02±2.79
[39]	Lasso Regression	own data	>233	demographic, frequency, original, derivatives (1–4)	unknown	6.9±9.0
	Lasso Regression		>233	frequency, original, derivatives (1–4)	unknown	7.8±10.4
[40]	SVR	Queensland	9	original	yes	11.6415±8.2022
[41]	linear regression	own data	21	original, frequency	yes	3.90±5.37
[42]	combinatorial ANN	UCI (MIMIC II), own data	46	original, second derivative	no	6.85±4.47
[43]	SVR	own data	12	demographic, original	no	4.9±4.9
[44]	SVR	Queensland	18	original	no	4.63±7.43
[45]	linear regression	own data	unknown	unknown	yes	7.66

**Table 2:** Description of used features. The ‘Beat’ column indicates whether the original beat (‘original’) or the sum of the kernels (‘reconstructed’) was used to extract the feature. All features but b/a are from the work of Hu et al. [13].

Category	Feature	Description	Beat
PWD	P1	amplitude of first kernel	reconstructed
	P2	amplitude of second kernel	reconstructed
	T1	mode of first kernel	reconstructed
	T2	mode of second kernel	reconstructed
	W1	width of first kernel	reconstructed
	W2	width of second kernel	reconstructed
Second Derivative	b/a	quotient of amplitudes of *b* and *a* wave of second derivative	reconstructed
Statistical Features	SD	standard deviation of pulse wave	reconstructed
	kurt	kurtosis of pulse wave	reconstructed
	skew	skewness of pulse wave	reconstructed
	PW	width of pulse wave	reconstructed
Frequency Features	Freq0	fundamental frequency	original
	Freq1	frequency of first harmonic	original
	Freq2	frequency of second harmonic	original
	Freq3	frequency of third harmonic	original

**Table 3:** Results of the prediction of our model for the all datasets combined and the single datasets separately.

Metric	Whole Dataset	CPT	Queensland	PPG-BP
MAE	9.456mmHg	5.799mmHg	12.981mmHg	18.593mmHg
ME	0.421mmHg	1.925mmHg	−1.137mmHg	9.471mmHg
SDE	13.195mmHg	7.481mmHg	16.799mmHg	22.677mmHg
*r*	0.730	0.796	0.372	0.274

**Table 4:** BP characteristics for the test and training set of the single datasets separately.

Metric	Split	CPT	Queensland	PPG-BP
maximum	training	211mmHg	206mmHg	182mmHg
	test	187mmHg	144mmHg	182mmHg
minimum	training	79mmHg	69mmHg	82mmHg
	test	98mmHg	64mmHg	84mmHg
mean	training	128.849mmHg	113.762mmHg	127.983mmHg
	test	130.487mmHg	106.377mmHg	131.281mmHg
standard deviation	training	16.908mmHg	25.713mmHg	21.501mmHg
	test	12.900mmHg	17.240mmHg	23.354mmHg

## Data Availability

Publicly available datasets were analyzed in this study. The PPG-BP data can be found here: https://figshare.com/articles/dataset/PPG-BP_Database_zip/5459299 (accessed on 22 July 2022). The Queensland data can be found here: https://outbox.eait.uq.edu.au/uqdliu3/uqvitalsignsdataset/index.html (accessed on 22 July 2022).
